# Identification and validation of a genetic risk signature associated with prognosis in clear-cell renal cell carcinoma patients

**DOI:** 10.1097/MD.0000000000034582

**Published:** 2023-08-04

**Authors:** Meiqin Lian, Yueyuan Feng, Zhenyu Wu, Zhonghong Zheng, Huanhuan Liu, Jian Li, Huixia Yu, Changlin Lian

**Affiliations:** a Blood purification center, The First Affiliated Hospital of Jinan University, Guangzhou, Guangdong, China; b Cancer Hospital, The First People’s Hospital of Foshan, Foshan, Guangdong, China; c Department of Urology, The First People’s Hospital of Foshan, Foshan, Guangdong, China; d Minimally Invasive Interventional Therapy, Sun Yat-Sen University Cancer Center, Guangzhou, Guangdong, China; e Department of Nephrology, The First Affiliated Hospital of Jinan University, Guangzhou, Guangdong, China; f Department of Neurology, The First People’s Hospital of Foshan, Foshan, Guangdong, China.

**Keywords:** clear-cell renal cell carcinoma, immune infiltration, prognostic-related genetic risk signatures, RNA-seq

## Abstract

Clear-cell renal cell carcinoma (ccRCC) is the most common subtype of renal cell carcinoma (RCC), which exhibits great variability in the prognosis of patients. Endoplasmic reticulum stress (ERS) is a persistent state triggered by disruption of endoplasmic reticulum (ER) homeostasis, which has been shown to control multiple pro-tumor-associated pathways in malignant cells while dynamically reprogramming immune cell function. This study aimed to identify ERS-related genetic risk signatures (ERSGRS) to ameliorate survival prediction in ccRCC patients. In this study, we adopted differentially expressed genes (DEGs) from the Cancer Genome Atlas (TCGA) and constructed ERSGRS with independent prognostic significance by least absolute shrinkage and selection operator (LASSO) regression. After separation of patients based on risk score, survival analysis showed that low-risk patients had longer overall survival (OS) than high-risk patients, and receiver operating characteristic (ROC) curve analysis confirmed the strong predictive ability of ERSGRS. Meanwhile, the tumor microenvironment (TME) of the high-risk group demonstrated an immunosuppressive phenotype, with more infiltration of regulatory T cells (Tregs) and macrophages. The TME in the low-risk group had a stronger potential for anti-tumor immunity. Overall, the ERSGRS could be a valuable predictive tool for ccRCC prognosis.

## 1. Introduction

Renal cell carcinoma (RCC) is a malignant tumor of the urinary system with obvious genetic characteristics.^[1]^ Until 2021, RCC ranks sixth in men and tenth in women, accounting for 5% and 3% of all tumor diagnoses, respectively.^[2]^ RCC incidence and mortality vary significantly across countries.^[3]^ Clear-cell renal cell carcinoma (ccRCC) is the most common type of RCC, characterized by genetic mutations in factors controlling signaling pathways, resulting in metabolic dysregulation, enhanced angiogenesis, intra-tumoral heterogeneity, and the tumor immune microenvironment Change.^[4,5]^ Early ccRCC tends to have a better prognosis, but the mortality rate will gradually increase in the late stage. Tyrosine kinase targeting vascular endothelial growth factor receptor pathway before immune checkpoint inhibitors (ICIs) that block the programmed cell death protein 1/programmed death-ligand 1 or cytotoxic T-lymphocyte-associated protein 4 T cell inhibitory receptors become clinically available inhibitors are the standard of treatment of ccRCC.^[6]^ With the clinical application of ICIs, they have been shown to be very effective in this disease and are now considered the standard in treatment-naïve and pretreatment patients.^[7,8]^ However, the efficacy of ICIs varies widely among patients with advanced ccRCC. We urgently need to construct a robust genetic signature in order to better identify and predict the response of ccRCC patients to ICIs and adjust the treatment plan timely. Recently several robust polygenic signatures have been developed to assess response to immunotherapy or prognosis in ccRCC patients. Yin et al established a prognostic model for cell cycle-related genes.^[9]^ Xu F et al constructed prognostic models for 8 glycolysis-related gene signatures.^[10]^ Guo X et al constructed a prognostic gene signature by the metabolism-related genes RRM2 and ALDH6A1.^[11]^ These unique genetic characteristics have unique predictive value. This indicates that finding effective tumor genetic information features is of great significance not only for early diagnosis and prognosis, but also for finding new therapeutic targets.

The endoplasmic reticulum (ER) is an organelle for protein processing, modification, and folding, which plays an important role in determining cellular function, fate, and survival.^[12]^ Under the influence of various unfavorable factors, such as gene mutation, hypoxia, nutrient deficiency and other unfavorable microenvironment can disrupt the homeostasis of ER in malignant cells and stromal cells.^[13]^ Therefore, endoplasmic reticulum stress (ERS) such as unfolded protein response and calcium ion disturbance will occur in cancer cells.^[14]^ It has been demonstrated in multiple cancer studies that ERS can control multiple tumor-promoting properties of cancer cells while dynamically reprogramming the function of innate and adaptive immune cells.^[15,16]^ ERS-related genes may interact to determine the fate of cancer cells.^[13]^ Currently, ERS studies related to ccRCC are relatively lacking. Therefore, we hope that studying the relationship between ERS-related genes and ccRCC prognosis will help to formulate effective cancer treatment and prevention strategies.

In our work, we constructed a 9 gene ERS-associated genetic risk signature (ERSGRS) to predict the prognosis of ccRCC patients based on the Cancer Genome Atlas (TCGA)-ccRCC by least absolute shrinkage and selection operator (LASSO). The ERSGRS was constructed to divided patients into low-risk and high-risk groups. Receiver operating characteristic (ROC) curve analysis confirmed the predictive ability of ERSGRS. We hope that the ERSGRS can provide some guidance for clinical judgment and individualized treatment.

## 2. Materials and methods

### 2.1. Sample sources and processing

We obtained information on ERS-related genes and corresponding clinical data of patients with ccRCC from the TCGA (https://cancergenome.nih.gov/). We set | log2FC | >0.5 and false discovery rate < 0.05 as thresholds to recognize the differentially expressed genes (DEGs) and ERS-related genes based on the limma R package. Univariate Cox regression was performed on ERS-related genes and clinical data to identify ERSGRS. We used LASSO regression analysis to identify ERSGRS closely associated with overall survival (OS).

### 2.2. Construction of the risk score model

The risk score model consisting of ERSGRS expression levels was established as follows: Risk score = (0.1805 × expression level of RCN3) + (0.4392 × expression level of CASP4) + (−0.0873 × expression level of SCAMP5) + (0.2436 × expression level of CHAC1) + (0.1242 × expression level of TRIB3) + (0.0098 × expression level of TNFRSF10B) + (0.2201 × expression level of DDX11) + (0.1920 × expression level of PDIA2) + (0.1112 × expression level of PLA2G6) (Table 1). The risk score for each patient was calculated according to the equation. In addition, we divided patients into high-risk and low-risk groups based on the median risk score. The Kaplan–Meier survival curve showed a prognostic difference between high-risk and low-risk patients. We conducted a subgroup analysis to further validate the model.

**Table 1:** The prognostic significance of the 9-genes signature.

ERS-related gene	Coef
RCN3	0.180582450
CASP4	0.439298417
SCAMP5	−0.087362308
CHAC1	0.243608039
TRIB3	0.124251716
TNFRSF10B	0.009817096
DDX11	0.220139887
PDIA2	0.192021879
PLA2G6	0.111288569

ERS = endoplasmic reticulum stress.

### 2.3. Establishment and evaluation of the prognostic model

The risk score and clinical characteristics such as age, gender, clinical stage, and TNM stage were used in the prognostic model. A nomogram was established based on the results of multivariate Cox regression to predict each patient 3- and 5-year OS. We used calibration plots generated by the rms package to evaluate the properties of the nomogram. We further assessed the accuracy of the nomogram by performing ROC curve analysis to obtain area under the ROC curve (AUCs). Then, the calibration curve and DCA were conducted to evaluate the model.^[17]^

### 2.4. Gene set enrichment analysis (GSEA)

We performed GSEA analyses on the genes differentially expressed between the high-risk and low-risk groups. The functions were derived by analyzing the gene set between 2 biological states.

### 2.5. Immune landscape analysis

We compared the low- and high-risk groups’ immune cell abundance based on TIMER, CIBERSORT, CIBERSORT-ABS, QUANTISEQ, MCPCOUNTRE and XCELL.^[18,19]^ We use the single sample GSEA to compare the low- and high-risk groups’ immune function. Using Spearmen correlation test, we explored the expression of immune cell inhibitory receptors and ligands in the low- and high-risk groups.

### 2.6. Statistical analysis

To determine the relationship between prognosis-related genes and patients with ccRCC, we performed Pearson correlation analysis. To compare differences between categorical and continuous variables, chi-square and t-tests were used, respectively. Univariate Cox regression, multivariate Cox regression, LASSO regression analysis and Kaplan–Meier method were performed to determine the best prognostic factors. The OS of each group was evaluated by Kaplan–Meier method, and the difference between groups was evaluated by log-rank test. Results were considered statistically significant at a 2-tailed *P* < .05.

## 3. Result

### 3.1. Identification of ERS-related DEGs

**Figure 1.**
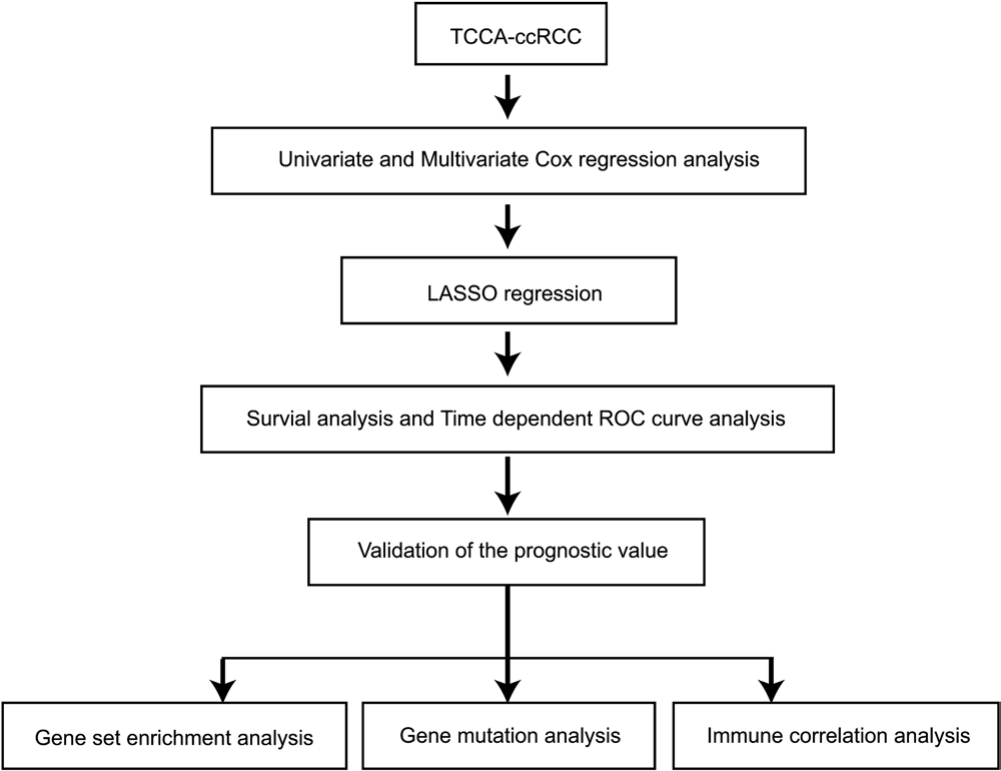
The design and procedure of our study are shown in the flow chart.

**Figure 2.**
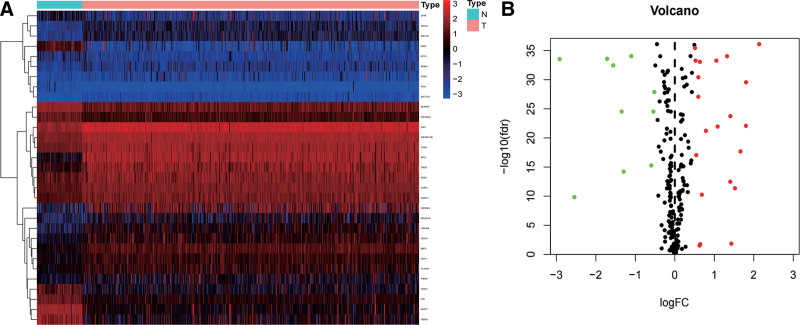
Identification of ERS-related DEG. (A) The heat map shows the ERS-related genes in normal tissues and ccRCC tumor tissues. (B) Volcano plot of the differentially expressed ERS-related genes: Red dots represent upregulated genes and green dots represent downregulated genes. Black dots indicate similarly expressed genes. ccRCC = clear-cell renal cell carcinoma, ERS = endoplasmic reticulum stress.

The design of our study is shown in Figure 1. To predict the prognosis of this disease, we compared the ERS-related genes expression between normal and tumor tissue from TCGA database by the limma R package. As a result, based on the criteria of |log2FC| > 0.5 and false discovery rate < 0.05, a total of 31 differentially expressed ERS-related genes were obtained, of which 21 genes were upregulated and 10 genes were downregulated. The distribution of these differently expressed ERS-related genes is shown in Figure 2A and B.

### 3.2. Construction of prognosis-associated signature

**Figure 3.**
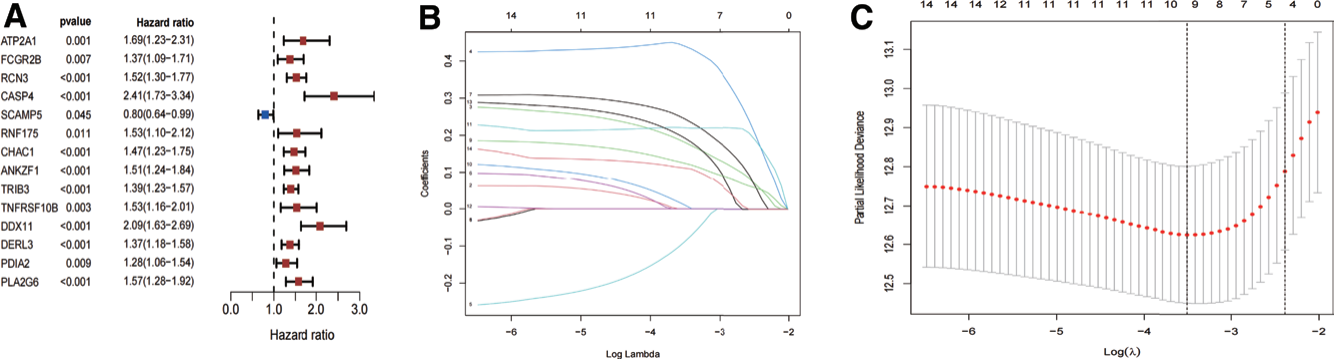
Construction of prognosis-associated signature. (A) Identification of 14 ERS-related genes in significant association with OS by univariate Cox regression analysis. (B and C) Candidate the ERS-related genes for constructing predictive signatures were screened by LASSO regression analysis. ERS = endoplasmic reticulum stress, LASSO = least absolute shrinkage and selection operator, OS = overall survival.

To explore the prognostic value of the ERS-related genes in renal cancer progression, we performed univariate Cox regression analysis to examine the potential relationships between the expression levels of 31 ERS-related genes and patient’ OS. Results demonstrated that 14 ERS-related genes were significantly associated with OS in ccRCC patients (*P* < .01) (Fig. 3A). Among those, ATP2A1, FCGR2B, RCN3, CASP4, RNF175, CHAC1, ANKZF1, TRIB3, TNFRSF10B, DDX11, DERL3, PDIA2, and PLA2G6 were considered as risk genes (HR > 1), while SCAMP5 was considered as protective genes (HR < 1) (Fig. 3A). We further screened LASSO regression on the above-mentioned 14 ERS-related genes to identify the most optimal risk score model for predicting survival in ccRCC patients (Fig. 3B and C). Finally, RCN3, CASP4, SCAMP5, CHAC1, TRIB3, TNFRSF10B, DDX11, PDIA2, and PLA2G6 were kept as target genes, and their respective coefficients were calculated to construct an ERS-related genetic signature (ERSGRS) to predict ccRCC prognosis.

### 3.3. Validation of the ERSGRS and performance analysis

**Figure 4.**
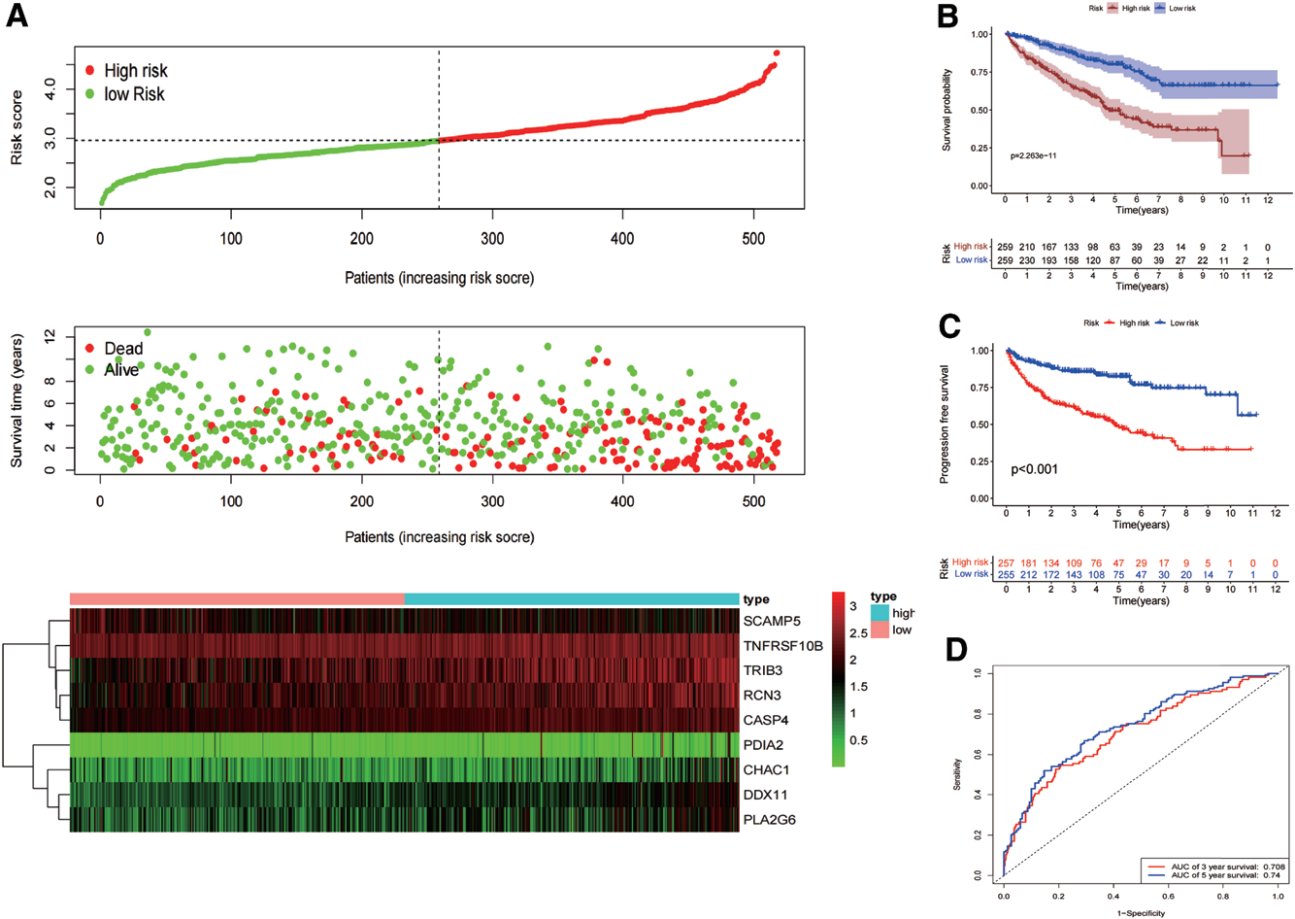
Validation of the ERSGRS and performance analysis. (A) The upper panel represents the distribution of risk scores for each patient, the middle panel shows the distribution of patients with increased risk values, and the lower panel represents the expression of 9 ERS-related genes between high-risk and low-risk groups. (B and C) Kaplan–Meier survival curves showing the OS and the PFS in high-risk and low-risk groups, respectively. (D) ROC curve analysis of the risk score. ERS = endoplasmic reticulum stress, ERSGRS = endoplasmic reticulum stress-related genetic risk signature, OS = overall survival, PFS = progression free survival.

We constructed the OS prognostic signature based on the expression of the 9 target genes and their prognostic coefficients using the following formula: Risk score = (0.1805 × expression level of RCN3) + (0.4392 × expression level of CASP4) + (−0.0873 × expression level of SCAMP5) + (0.2436 × expression level of CHAC1) + (0.1242 × expression level of TRIB3) + (0.0098 × expression level of TNFRSF10B) + (0.2201 × expression level of DDX11) + (0.1920 × expression level of PDIA2) + (0.1112 × expression level of PLA2G6) (Table 1). We assessed survival time, survival status and expression of 9 ERS-related genes according to the risk score (Fig. 4A). Moreover, according to the median risk score (50%), 257 and 255 ccRCC patients were sorted into a high-risk group and a low-risk group, respectively. Afterwards, the Kaplan–Meier curve displayed that the OS of the low-risk group was better than that of the high-risk group (Fig. 4B) and the progression free survival of the low-risk group was better than that of the high-risk group in the TCGA datasets (Fig. 4C). In addition, the ROC curve was applied to evaluate the accuracy of the ERS-related genes signature. The AUC was 0.708 and 0.740 for survival in 3- and 5-year, respectively (Fig. 4D).

### 3.4. Independent prognostic value of ERSGRS

**Figure 5.**
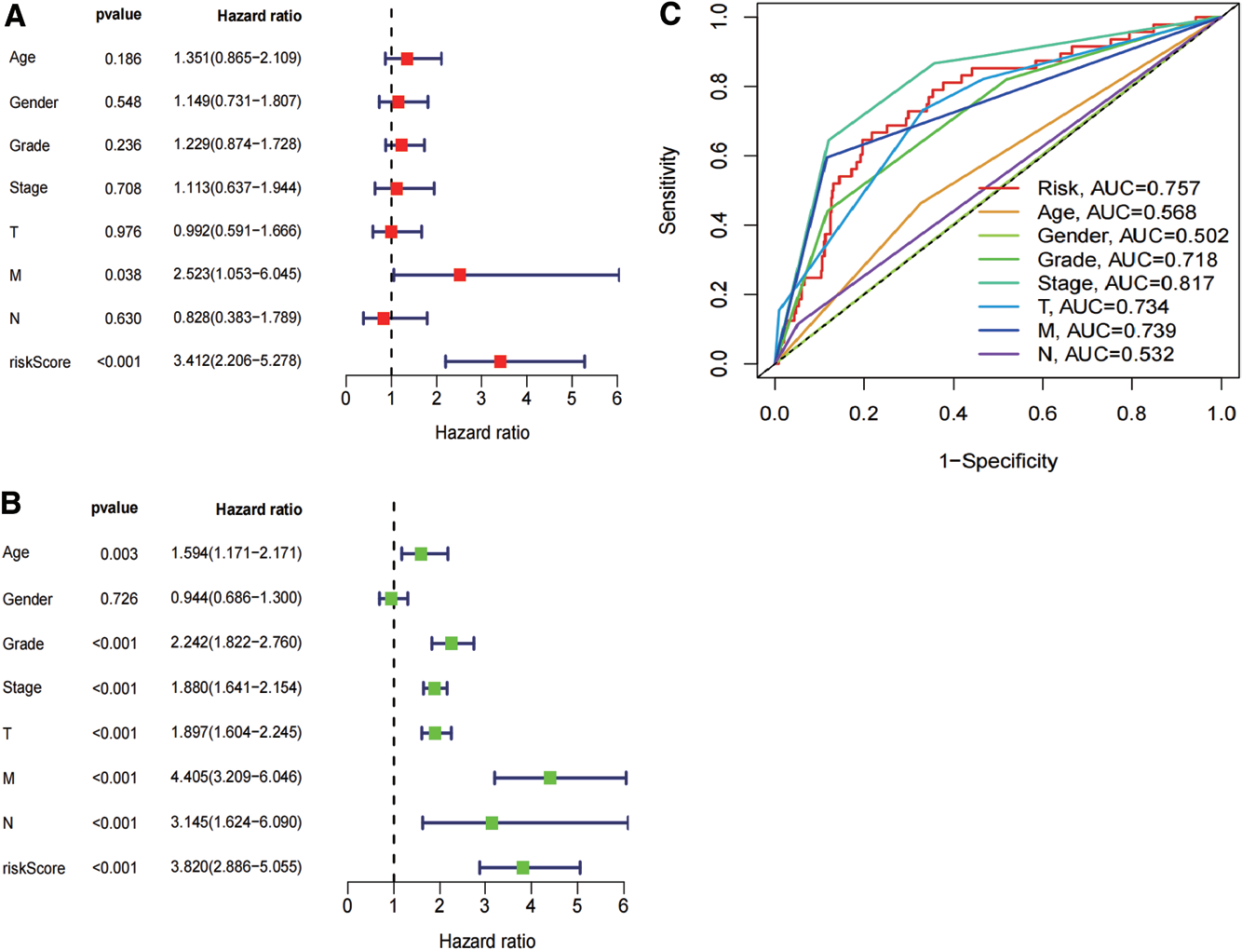
Independent prognostic value of ERSGRS. (A and B) Analysis of the Univariate and Multivariate Cox regression analysis revealed risk score was strongly associated with OS in the TCGA dataset. (C) Time dependent ROC curves of risk score, age, gender, grade, stage, T stage, M stage and N stage. ERSGRS = endoplasmic reticulum stress-related genetic risk signature, OS = overall survival, ROC = receiver operating characteristic curve, TCGA = the Cancer Genome Atlas.

We performed univariate and multivariate Cox regression analyses to further determine whether the prognostic signature could serve as an independent prognostic factor. Univariate analysis revealed that risk score was significantly associated with OS (Fig. 5A). After adjusting for other confounders, subsequent results showed that age (*P* = .003), grade (*P* < .001), stage (*P* < .001), T stage (*P* < .001), M stage (*P* < .001), N stage (*P* < .001) and risk score (*P* < .001) were significantly correlated with OS in multivariate analyses (Fig. 5B). Furthermore, the AUCs for gene signatures exhibited excellent predictive ability for survival compared to clinicopathological factors (Fig. 5C). These data indicate that ERSGRS was a highly reliable genetic signature for ccRCC patients.

### 3.5. GSEA of high- and low-risk group

**Figure 6.**
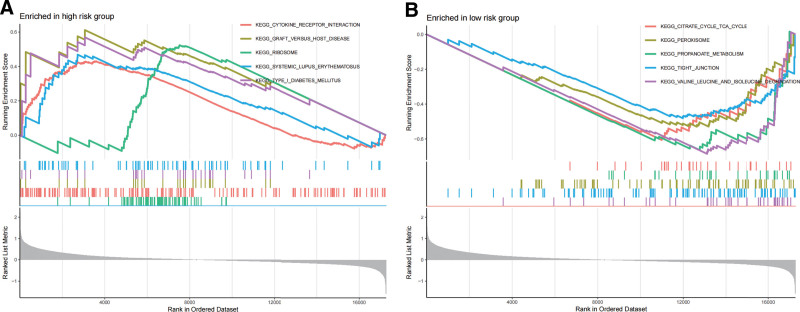
GSEA of high-risk group and low-risk group. (A) The significant pathways were enriched in the high-risk group by performing the GSEA analysis. (B) The significant pathways were enriched in the low-risk group by performing the GSEA analysis. GSEA = gene set enrichment analysis.

To further explore the function between the high- and low-risk group, we performed GSEA of the DEGs between the low- and high-risk group. The results showed that cytokine receptor interaction, graft versus host disease, ribosome, systemic lupus erythematosus, and type I diabetes mellitus were enriched in the high-risk group (Fig. 6A), while citrate cycle tca cycle, peroxisome, propanoate metabolism, tight junction, valine leucine and isoleucine degradation were enriched in the low-risk group (Fig. 6B). GSEA indicated that the ERSGRS were associated with these oncogenic pathways. However, the specific signaling pathway needs to be further explored.

### 3.6. Immune landscape in the high- and low-risk groups

**Figure 7.**
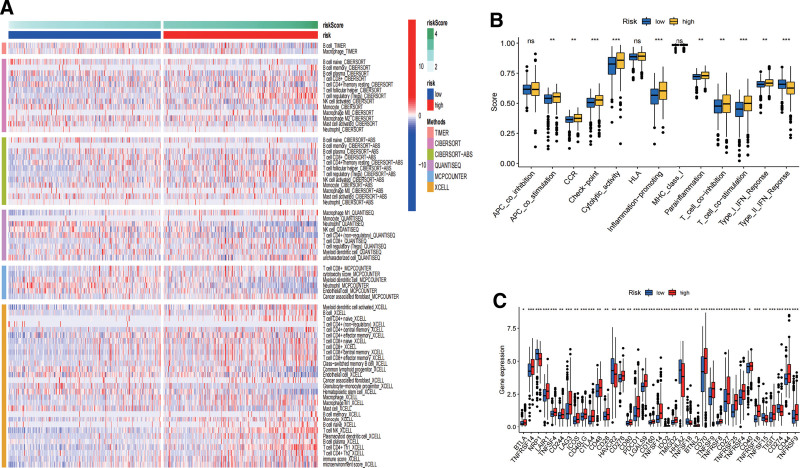
Immune landscape in the high- and low-risk groups. (A) Heatmap showing immune response infiltration in the high- and low-risk groups. (B) ssGSEA showed activation of immune-related pathways in the high- and low-risk groups. (C) Box-plot showing differential expression of immune checkpoints and antigen presentation in the high- and low-risk groups. ssGSEA = single sample Gene Set Enrichment Analysis.

The high-risk and low-risk groups displayed significant differences in the distribution of tumor-invading immune cells. Particularly, compared to the tumor microenvironment (TME) of the low-risk group, the TME of the high-risk group included considerably more regulatory cells (Tregs), M1 macrophages cell, natural killer cell, B cell, activated CD8 and CD4 T cell (Fig. 7A). The function of CD4 + and CD8 + effector T cells, natural killer cells, M1 macrophages cell, and B cell may all be inhibited by Tregs in a number of ways, according to the available research, which results in an ineffective immune response and a poor prognosis.^[20,21]^ The low-risk group had higher levels of infiltrating endothelial cells and neutrophil (Fig. 7A). Antigen-presenting cells called endothelial cells have drawn more interest recently. They contribute to the recognition, preparation, and presentation of antigens as well as the start of the T cell-mediated immune response. Neutrophils are a type of phagocyte and neutrophil-derived ROS, such as hydrogen peroxide and nitric oxide, are cytotoxic to cancer cells, and neutrophils in early-stage tumors acquire an antigen-presenting signature associated with their ability to stimulate T-cell responses.^[22]^ According to single sample GSEA, with the exception of Type II IFN, immune-related pathways were more active in the high-risk group (Fig. 7B). Type II IFN is of great value in the study of cancer immunotherapy due to its ability to prevent tumor growth.^[23]^ Furthermore, there was a statistically significant difference between the high-risk and low-risk groups in terms of tumor immune response (Fig. 7C). BTLA, TNFRSF14, LAG3, LAIR1, CTLA4, PDCD1, LGALS9, CD160, BTNL2, TNFRSF8, TIGIT, TNFSF18, TMIGD2, NRP1, CD44, TNFRSF18, HHLA2, HAVCR2, and CD274 as markers of immune checkpoint activity were selected for analysis.^[24–26]^ TNFSF4, CD244, CD40LG, CD48, CD276, CD80, TNFSF14, IDO2, ICOS, CD27, CD28, CD70, TNFSF9, TNFRSF9, TNFRSF25, TNFSF15, TNFRSF4, and CD40 as markers of immune activity were selected for analysis.^[27–29]^ Compared with the low-risk group, the high-risk group had elevated levels of BTLA, TNFRSF14, LAIR1, TNFSF4, CD244, LAG3, ICOS, CD40LG, CTLA4, CD48, CD28, CD276, CD80, PDCD1, LGALS9, CD160, TNFSF14, IDO2, TMIGD2, BTNL2, CD70, TNFSF9, TNFRSF8, CD27, TNFRSF25, TNFRSF4, CD40, TNFRSF18, TIGIT, CD44 and TNFRSF9 (Fig. 7C). These data indicate that despite a richer immune cell infiltration and a more active immune response in patients in the high-risk group, there is immunosuppression. Patients in the low-risk group had stronger potential for anti-tumor immunity. This may be the main reason for the better prognosis of patients in the low-risk group than in the higher-risk group.

## 4. Discussion

The ER serves as the central organelle of the cell and is responsible for regulating protein synthesis, folding, modification and trafficking, and lipid metabolism.^[12]^ But when affected by events outside or inside the cell, ER homeostasis is disrupted and accumulation of misfolded or unfolded proteins occurs. Continued accumulation can trigger an ERS state.^[13]^ This state confers greater tumorigenic, metastatic, and drug-resistant capabilities on malignant cells, and may disrupt cellular homeostasis leading to cell death.^[13]^ ERS-related pathways have emerged as key regulators of tumor progression.^[15,16]^ ERS-related genes can serve as a reliable genetic signature to predict tumor progression.

In this study, we constructed an ERSGRS composed of RCN3, CASP4, SCAMP5, CHAC1, TRIB3, TNFRSF10B, DDX11, PDIA2, and PLA2G6 by LASSP to predict the prognosis of ccRCC patients. Among these prognostic genes, many genes remain to be further studied. For example, RCN3 can regulate protein kinase B (PKB) signaling to activate anti-apoptotic mechanisms, glucose metabolism, and protein synthesis, thereby promoting cell growth and proliferation.^[30]^ CASP4 plays a central role in the execution phase of apoptosis and participates in the signaling pathways of apoptosis, necrosis and inflammation.^[31,32]^ SCAMP5 is involved in the upregulation of cytokine production; regulation of vesicle-mediated trafficking; and response to ERS.^[33]^ CHAC1 is a ferroptosis-related gene, and its increased expression indicates an increased risk of cancer recurrence in patients with breast and gastric cancer.^[34,35]^ TRIB3 reduces CD8 T cell infiltration and induces immune escape by inhibiting the STAT1-CXCL10 axis in colorectal cancer.^[36]^ TRIB3 promotes the development of MYC-associated lymphoma by inhibiting UBE3B-mediated degradation of MYC.^[37]^ In addition, TRIB3 mediates ERS-induced β-cell apoptosis via the NF-κB pathway.^[38]^ TNFRSF10B can be activated by tumor necrosis factor-related apoptosis-inducing ligands and transduce apoptosis signals.^[39]^ DDX11 is involved in various functions of genome stability, including DNA replication, DNA repair and heterochromatin organization, and ribosomal RNA synthesis.^[40]^ PDIA2 plays a role in the folding of nascent proteins in the ER to regulate signaling.^[41]^ PLA2G6 can mediate apoptosis and premature degeneration of dopaminergic neurons in the substantia nigra.^[42,43]^ Meanwhile, ROC-AUC estimates indicated that the prognostic features exhibited by ERSGRS performed favorably. Subsequent clinical application analysis further demonstrated that the signature could accurately distinguish the prognostic outcomes of high-risk and low-risk patient populations.

We used the expression and risk coefficient of the ERSGRS gene to divide ccRCC patients into low-risk and high-risk groups. We found that high-risk groups had poorer outcomes than low-risk groups and many signaling pathways related to the citric acid cycle tca cycle, peroxisome, propanoate metabolism, tight junction, valine leucine and isoleucine degradation were significantly activated in high-risk groups. Furthermore, we found clear differences in the extent of immune cell infiltration in TME between high-risk and low-risk groups. Although there are abundant immune cells in the TME of high-risk patients, existing studies have shown that Tregs can affect their function and induce immune evasion of tumor cells.^[21]^ And we found that compared with the low-risk group, the expression of immune checkpoint-related genes in the high-risk group was significantly increased, which is also one of the reasons for the immune evasion of tumor cells. However, we found higher levels of infiltrating endothelial cells and neutrophils and Type II IFN-related immune pathways were more active in the low-risk group. Compared with the high-risk group, the expression of immune checkpoint activation genes was lower in the low-risk group. This explains the better prognosis of patients in the low-risk group relative to the high-risk group.

This study also has limitations. The present study lacks in vivo and in vitro experiments to support. Further in vitro and in vivo experimental validation is necessary to elucidate the mechanisms underlying the predicted metabolic gene regulation. Second, we do not have sequencing data before and after clinical treatment. If supported by relevant data, the accuracy of ERSGRS in predicting prognosis can be improved. This is conducive to the further optimization of the ERSGRS model. In the future, we will do further research in the above-mentioned aspects.

In conclusion, we extracted 9 ERS-related genes and constructed a novel prognostic signature that could accurately and independently predict the prognosis of ccRCC patients. These studies indicate that the ERS-related genes signatures identified may provide guidance for clinical judgment and personalized treatment of ccRCC.

## Author contributions

**Data curation:** Huixia Yu, Meiqin Lian, Yueyuan Feng, Zhenyu Wu.

**Formal analysis:** Meiqin Lian, Yueyuan Feng.

**Project administration:** Huixia Yu, Changlin Lian, Huanhuan Liu, Jian Li.

**Supervision:** Huixia Yu, Changlin Lian.

**Writing – original draft:** Huixia Yu, Yueyuan Feng, Zhonghong Zheng, Huanhuan Liu, Jian Li.

**Writing – review & editing:** Huixia Yu, Yueyuan Feng, Zhenyu Wu, Zhonghong Zheng, Huanhuan Liu.
